# Resistance exercise combined with protein supplementation for skeletal muscle mass in people with pancreatic cancer undergoing neoadjuvant chemotherapy: Study protocol for the REBUILD trial

**DOI:** 10.1371/journal.pone.0322192

**Published:** 2025-05-02

**Authors:** Paola Gonzalo-Encabo, John Gardiner, Mary K. Norris, Rebekah L. Wilson, Amber J. Normann, Danny Nguyen, Nathan Parker, Darryl Tjogas, Lauren K. Brais, Jeffrey A. Meyerhardt, Michael H. Rosenthal, Brian M. Wolpin, Hajime Uno, Christina M. Dieli-Conwright

**Affiliations:** 1 Department of Medical Oncology, Dana-Farber Cancer Institute, Boston, Massachusetts, United States of America; 2 Department of Medicine, Harvard Medical School, Boston, Massachusetts, United States of America; 3 Departamento de Ciencias Biomédicas, Área de Educación Física y Deportiva, Facultad de Medicina y Ciencias de la Salud, Universidad de Alcalá, Madrid, España; 4 Department of Health Sciences, Boston University, Boston, Massachusetts, United States of America; 5 Department of Health Outcomes and Behavior, Moffitt Cancer Center, Tampa, Florida, United States of America; 6 Department of Imaging, Dana-Farber Cancer Institute, Boston Massachusetts, United States of America; 7 Department of Radiology, Brigham and Women’s Hospital, Boston, Massachusetts, United States of America; McMaster University, CANADA

## Abstract

**Background:**

Pancreatic cancer patients’ prognosis may be limited by two conditions, cachexia and sarcopenia. Resistance exercise and protein supplementation are safe non-pharmacological strategies that may increase or preserve skeletal muscle mass within this population. Therefore, the primary aim of this study is to examine the feasibility of a home-based virtually supervised resistance exercise intervention, with or without protein supplementation in pancreatic cancer patients initiating neoadjuvant chemotherapy. This intervention may also maintain skeletal muscle mass and improve plasma biomarkers associated with muscle tissue wasting, physical function and psychological measures.

**Methods:**

We aim to recruit 45 patients with locally advanced pancreatic cancer initiating neoadjuvant chemotherapy. Patients will be randomized to receive either Resistance Exercise (RE) (n = 15), Resistance Exercise and Protein Supplementation (RE + PS) (n = 15), or Attention Control (AC) (n = 15). Patients randomized to RE or RE + PS will receive 16-weeks of home-based virtually supervised resistance exercise. The AC will receive a 16-week stretching program. Primary and secondary outcomes will be measured at baseline and after 16 weeks during study visits.

**Discussion:**

The REBUILD trial is the first randomized controlled trial that combines resistance exercise with daily protein supplementation during neoadjuvant chemotherapy in pancreatic cancer patients. Our novel home-based virtually supervised exercise intervention seeks to mitigate barriers to participation in this vulnerable population. Furthermore, results of this trial will address important research gaps associated with pancreatic cancer-related cachexia, a condition closely connected with poor prognosis and mortality.

## Introduction

Pancreatic ductal adenocarcinoma is one of the most fatal and aggressive cancer diseases, with an estimated 5-year survival rate of 13%. [1] Pancreatic cancer is projected to become the second leading cause of cancer death by 2030 in the U.S., given the demographic shift towards an increasingly aging population. [2] The current standard of care in patients with resectable pancreatic ductal adenocarcinoma is surgery and systemic chemotherapy. However, chemotherapy and the disease itself can induce muscle wasting and weakness leading to a diagnosis of cachexia and/or sarcopenia. [3] Cachexia is a tissue-wasting syndrome characterized by severe weight loss, typically defined as an unintended loss of more than 10% of pre-illness stable weight. [4] Sarcopenia, on the other hand, involves the progressive loss of skeletal muscle mass, strength, and function. [5] Both conditions are associated with reduced physical function, poor responses to treatment, and decreased survival rates among patients with pancreatic ductal adenocarcinoma. [6–9] Nonetheless, no systemic strategies are provided in current standard of care to prevent potential muscle wasting for patients with pancreatic cancer.

Resistance exercise and protein supplementation are safe non-pharmacological strategies that may increase or preserve skeletal muscle mass in various cancer settings, with most evidence in prostate cancer [10,11] and breast cancer. [12–14] Indeed, resistance training is recommended as an essential component for sarcopenia and cancer cachexia treatment. [10,15] While aerobic exercise provides substantial cardiovascular and systemic benefits, it is less effective in directly addressing sarcopenia related outcomes. [10,15] Pancreatic cancer patients are largely underrepresented in exercise and nutrition interventions. Exercise-induced benefits in pancreatic cancer patients have been previously reported for increasing physical activity participation, physical function, and quality of life, [16,17] with preliminary efficacy in improving skeletal muscle mass in case report studies. [18–20] The addition of protein supplementation is strongly associated with muscle mass maintenance or improvement in frail non-cancer populations. [21] Although numerous investigations support the use of post-exercise protein supplementation to enhance acute muscle protein synthesis in non-cancer population, [22] limited data attest to the long term effects of protein supplementation and resistance exercise with regards to its feasibility and effectiveness on skeletal muscle mass within the pancreatic cancer population. [18]

Thus, the ongoing REBUILD trial is a three-armed pilot randomized controlled trial designed to evaluate the feasibility of a home-based virtually supervised resistance exercise intervention, with or without protein supplementation, in pancreatic cancer patients initiating neoadjuvant chemotherapy. Our secondary aim is to examine the effects of home-based virtually supervised resistance exercise combined with or without protein supplementation on skeletal muscle mass, plasma biomarkers associated with muscle tissue wasting, physical function and psychosocial measures. We hypothesize that a home-based virtually supervised resistance exercise intervention with or without protein supplementation will be feasible, with at least 70% of exercise sessions completed and protein supplementation consumed among pancreatic cancer patients undergoing neoadjuvant chemotherapy. Furthermore, we hypothesize that a home-based virtually supervised resistance exercise combined with protein supplementation will elicit greater maintenance of skeletal muscle mass and improvements in plasma biomarkers associated with muscle wasting compared with resistance exercise alone and attention controls. Lastly, we hypothesize that resistance exercise alone or in combination with protein supplementation will improve physical function and psychosocial measures when compared to attention controls.

## 2. Materials and methods

### 2.1. Study design

The REBUILD trial is a single-center, three-armed, pilot randomized controlled trial (RCT) underway at Dana-Farber Cancer Institute (DFCI), Boston, Massachusetts. A total of 45 patients with locally advanced pancreatic cancer initiating FOLFIRINOX or gemcitabine/abraxane neoadjuvant chemotherapy are recruited from the Dana-Farber Gastrointestinal Cancer Center and randomized to either receive Resistance Exercise (RE) (n = 15), Resistance Exercise and Protein Supplementation (RE + PS) (n = 15), or Attention Control (n=15) (AC). The intervention groups participate in a home-based virtually supervised resistance exercise program three days per week with or without protein supplementation over the course of neoadjuvant chemotherapy (maximum 16-weeks). The intervention period was set at maximum 16 weeks to align with the common duration of the neoadjuvant chemotherapy prior to surgery in this population. The AC group receives a stretching program over the course of neoadjuvant chemotherapy and is offered the RE + PS program following completion of treatment and may begin the intervention within and up to 6 months post-surgical resection. Outcome measures are tested at baseline and within one week of intervention completion. Approval of the trial protocol was obtained from the DFCI Institutional Review Board (IRB# 21–506). Any amendments made are communicated to all appropriate authorities. This clinical trial is registered in ClinicalTrials.gov (NCT05356117).

### 2.2. Eligibility criteria

To be eligible to participate in this study, patients must meet the following inclusion criteria: 1) are ≥ 18 years of age, 2) non-metastatic pancreatic cancer initiating neoadjuvant chemotherapy, 3) speaks English or Spanish, 4) able to provide physician clearance to participate in the exercise program, 5) able to initiate a supervised exercise program, 5) currently participates in less than 60 minutes of structured moderate or vigorous exercise per week, 6) non-smoker (no smoking during previous 12 months), and 7) willing to travel to DFCI. Key exclusion criteria include: 1) metastatic disease, 2) uncontrolled illness including active infection, diabetes, hypertension, or thyroid disease, 3) history of musculoskeletal, cardiorespiratory, or neurological diseases that preclude the participation in moderate intensity exercise, 4) receiving other investigational agents and or other cancer directed treatments, 5) other active malignancies, and 6) regularly consuming protein supplementation.

### 2.3. Recruitment and screening

Patients are recruited through various recruitment strategies at the DFCI and regional site clinics. For potential patients identified through patient lists, we contact their medical oncology providers, and request permission to contact the patients to invite them to participate in the study. Potentially eligible patients are further screened by study staff by phone to confirm eligibility, which includes a short questionnaire to determine eligibility and completion of the Godin Leisure Time Questionnaire [23] to assist in determining the patient’s current exercise level. To confirm the patient’s health status and how this relates to undertaking an exercise program we use the Physical Activity Readiness Questionnaire. [24] Interested patients are then scheduled for a consent visit with a member of the study staff where we review the protocol and confirm interest by signing informed consent, either in person or electronically using e-consent. To successfully recruit and retain pancreatic cancer patients in an exercise clinical trial we employ several strategies including follow-up phone calls/emails after every visit, informative study brochures, randomization group flyers summarizing what to expect after testing, scheduling flexibility for testing or exercise training appointments, home-based virtually supervised exercise sessions, and transportation support resources (e.g. parking voucher, reimbursement for travel expenses, etc.).

### 2.4. Randomization and blinding

Upon completion of baseline testing, patients are randomly assigned to either RE, RE + PS or AC groups in an equal allocation ratio (1:1:1) using a permitted block design with varying block size. The study biostatistician (H.U.) prepared the randomization schema prior to trial start-up, and the randomization allocation was provided to staff via a web-based application (REDCap). Study investigators are blinded to the randomization process. The intervention allocation is not blinded to the study patients, interventionists, and outcome assessors due to the nature of exercise intervention.

### 2.5. Intervention

#### 2.5.1. Exercise intervention.

*2.5.1.1. Resistance exercise group (RE):*

The exercise intervention is home-based virtually supervised via Zoom. Patients complete three sessions per week for the duration of their neoadjuvant chemotherapy treatment with a maximum intervention duration of 16 weeks. The RE prescription consists of 2–3 sets of 8–12 repetitions at 70–85% estimated one-repetition maximum (1-RM) and/or at a rate of perceived exertion (RPE) of 6–7 on the Borg 10-scale. Session duration is approximately 60 minutes. Planned prescribed weight was calculated as a percent of estimated 1RM, which was tested at baseline via 10RM for each exercise. Initially, the weight was set at 70% of 1RM and increased by 5% every 2 weeks until week 8, reaching 85% of 1RM. From week 9 to 16, the prescribed weight increased where the highest tolerable weight for the intended repetitions and sets was used to gradually progress the weight. In the event a patient was not able to follow a linear progression due to injury or treatment-related side effects, the highest tolerable weight for the intended repetitions and sets was used. Exercises include chair squat, floor chest press, glute bridge, seated bent over row, lunges, and shoulder press. Exercises are tailored to the patients where the use of alternative exercises targeting similar muscle groups are incorporated depending on the health and physical status of the patients. Each exercise session begins with a 5-minute functional movement-based warm up and culminates with 5 minutes of stretching and 2 core exercises. Patients randomized to this group are provided with handheld weight-adjustable dumbbells and resistance bands. Patients keep all the exercise equipment after the intervention. Furthermore, an automated blood pressure cuff may be provided if study staff deem it necessary for patients’ safety.

*2.5.1.2. Resistance exercise combined with protein supplementation group (RE + PS):*

The same RE intervention described above is prescribed to the RE + PS group. Patients additionally receive and are asked to consume 40g per day of protein (Unjury, Middletown, CT) equating to 2 scoops of protein supplement, [25] aiming to increase their overall protein intake to 1.2–1.5 g/kg of ideal body weight per day, to align with the current protein intake guidelines for adults with chronic diseases and to maximize stimulation of skeletal muscle protein synthesis. [26] On the day of exercise (3 days per week) the patients are asked to consume the protein supplement immediately (within 30 minutes) after exercise. This has been reported as the optimum time to consume protein to enhance muscle protein synthesis. [21,27,28] Timing of protein supplementation intake on non-exercising days (4 days per week) is not dictated, although patients are recommended to consume the 40g dose with a meal, or over two meals (e.g., 20g at breakfast and 20g at lunch), and record time(s) of day consumed. [29] Patients are provided the protein supplement and a shaker bottle with recommendations on how the protein supplement may be prepared and consumed (e.g. with water, milk.).

*2.5.1.3. Attention control group*

This group performs a home-based program of the same stretches utilized in the two intervention groups. The stretching protocol consists of one set of 3–4 static stretching exercises held for 30 seconds and performed 3 days/week. As flexibility exercises are low intensity, low impact and low-volume, minimal caloric expenditure is expected to be incurred. To increase compliance [30] and aid in the standardization of the home-based stretching, [31] patients are provided a booklet of the flexibility exercises. Patients are shown how to use the booklet and instructed in the stretching exercises by study staff prior to the intervention. In addition, patients are asked to complete weekly records of flexibility compliance and physical activity performed outside the study. This group is offered the RE + PS intervention after their treatment period and may start anytime within and up to a time of 6 months post-surgery date. Given the intervention length is dictated by treatment, we offer an intervention that matches the length of the neoadjuvant chemotherapy regime they received during the initial intervention period up to a maximum of 16 weeks. Patients are offered the choice of daily protein supplement if they elect to partake in the RE intervention.

### 2.6. Outcome measures

**Fig 1:**
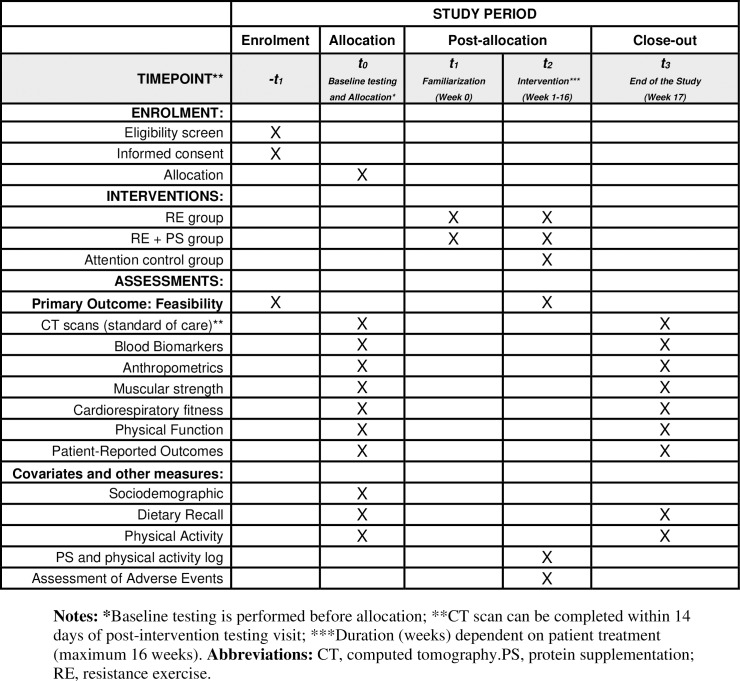
Spirit study timeline and data collection time points.

Testing is completed at baseline (Week 0) and within 7–10 days of intervention completion (maximum 16 weeks). (**Fig 1**) All measures are collected at all time points unless specified below. Baseline testing is performed before randomization. Patients are provided with monetary compensation for each testing timepoint attended, and parking validation for every visit to DFCI.

#### 2.6.1. Primary outcome.

*2.6.1.1. Feasibility*

Feasibility of the RE intervention The intervention is considered feasible if 1) patients attend ≥70% of the total prescribed number of exercise sessions (dependent on their treatment duration) [17,32] and 2) **≥**70% of sessions completed at the planned prescription (i.e., relative dose intensity ≥ 100%). The planned exercise volume (sets x reps x weight) is established prior to exercise session with the %1RM weights calculated from 10RM testing at baseline. However, in the event estimated 1RM is not available or the patient is not being adequately progressed during the intervention (i.e., weights become to easy to move relative to intended %1RM intensity), the lead exercise trainer would prescribe the highest tolerable weight based on pre-determined volume (sets x repetitions). A dose modification is defined as a reduction in sets, repetitions, and/or weight during the session that deviated from the planned volume, whereas a dose escalation is defined as an increase in sets, repetitions, and/or weight. Both dose modification and escalation were applied during the session at the discretion of the exercise trainer and reported as actual volume completed. Planned and actual volume for each resistance exercise were respectively calculated and summed to derive total planned and actual prescribed volume for each session, which were summed to obtain total planned and actual intervention volume. Exercise relative dose intensity is then calculated as the ratio of total actual resistance volume to total planned resistance volume. Reasons for missed sessions are documented throughout the study. [33] To account for missed sessions due to illness, family and work obligations, patients are given 6 exercise sessions to make-up at the end of their program if their treatment timeline permits. Feasibility of the RE ± PS intervention: The same criteria described above are used for the exercise portion. Additionally, patients are considered compliant and the PS intervention feasible if they consume ≥70% of the total prescribed doses of protein supplementation. Protein adherence is captured for the RE + PS group by asking patients during the supervised exercise sessions if they have been compliant with protein supplementation intake and recording on the exercise sessions data sheet.

#### 2.6.2. Secondary outcomes.

*2.6.2.1. Skeletal muscle mass*

Skeletal muscle mass is examined by standard-of-care CT scans. Skeletal muscle area is measured on axial CT images at the level of the L3 vertebral body using a validated, fully automated algorithm. [34] Skeletal muscle index (SMI) is calculated as the ratio of muscle area (cm^2^) to squared height (m^2^). This fully automated method estimates a patient’s body composition from an abdominal CT scan and has been validated across two large scale and diverse datasets. [35,36] Sarcopenia will be defined as index SMI < 38.5 cm^2^/m^2^ for women and < 52.4 cm^2^/m^2^ for men. [37]

*2.6.2.2. Cachexia-related blood biomarkers*

Fasting (12 hour fast) blood is drawn by a trained phlebotomist. For each patient, two 10 ml tubes are collected into EDTA and Serum tubes, along with one additional lavender topped tube (whole blood in EDTA). Samples are centrifuged and then aliquoted and stored at -80 degree C at DFCI for future measures. Blood samples are transported to the Wolpin lab for storage and batch-testing of plasma biomarkers occurs at DFCI for IL-6, MCP-1, TNF-RII, and branched chain amino acids by ELISA, and liquid chromatography tandem mass spectrometry.

#### 2.6.3. Tertiary outcomes.

*2.6.3.1. Anthropometrics*

BMI (kg/m^2^) is calculated from height and weight. A tape measure is used to obtain waist and hip circumference defined as the distance around the smallest part around the waist between the 12^th^ rib and iliac crest and the widest girth of the buttocks using the greater trochanter as a landmark, respectively. Body composition is assessed via bioelectrical impedance using a validated device (Tanita 780, Arlington Heights, IL).

*2.6.3.2. Muscular strength*

Upper body and lower body strength is measured using the 10-repetition maximum (10-RM) test using a machine chest press and leg press respectively using validated equations to calculate estimated 1-RM. [38,39] Following a warm-up, patients are given 5 attempts to reach the final 10-RM load with a 1–2-minute rest period between attempts. The 10-RM method has been validated in cancer populations with high to very high reliability. [40] Furthermore, for those randomized to the exercise group, the 1-RM is estimated from 10-RM muscle strength tests on the 6 exercises prescribed in the intervention to determinate exercise prescription: 1) chair squat, 2) chest press, 3) glute bridge, 4) row, 5) lunges, 6) shoulder press. Hand grip strength is measured using a hand-held dynamometer on the patient’s dominant hand. The subject is asked to grip the handle of the dynamometer with one hand using as much grip pressure as possible while holding for 5 seconds. The subject is asked to complete 3 grip strength attempts.

*2.6.3.3. Cardiorespiratory fitness*

Cardiorespiratory fitness is assessed by the 6-minute walk test (6MWT), a validated test in cancer survivors. [41] Patients are instructed to walk as quickly as possible without running on an indoor pre-measured walkway for 6 minutes. Distance achieved in 6 minutes is measured in meters.

*2.6.3.4. Blood pressure and resting heart rate*

Blood pressure (BP) and resting heart rate (HR) are measured using an automated device with an appropriately sized cuff (Omron BP 786, Lake Forest, IL) after the patient has sat quietly for 5 minutes while resting their arm on a table so the brachial artery is level with the heart.

*2.6.3.5. Physical function*

The short physical performance battery (SPPB) [42] includes three sections: 1) balance with feet together, semi tandem, and full tandem is held for up to 10 seconds (s) with no support; 2) usual gait speed over 4m is timed with two attempts given and the fastest time is recorded; and 3) the five-chair stand test is performed where the time to complete five chair sit-to-stands is recorded. A score is given for each section of the SPPB and a total summary score. The timed up and go (TUG) [43] test times how fast a patient could stand up from a chair, walk around a cone placed 3m away, and end in a seated position. Gait speed [44] over a 6m flat surface is assessed and the time to talk the course at a usual pace and a fast pace is recorded with two attempts given for each speed. The Margaria stair climb [45] is completed in a stairwell of ten stairs. Patients walk or run up the stairs as fast as they can. The time the patient takes from stair three to stair nine is recorded, with three attempts given after a familiarization practice, and the fastest time is used. Power is calculated from the stair climb using a validated equation. [45] The sit-to-stand [46] test times how many sit-to-stands a patient can perform in 30s from a seated chair position to a standing position with full hip extension. Only one attempt is given and the number of repetitions with correct technique are recorded.

*2.6.3.6. Patient-reported outcomes*

Health-related quality of life (QOL) is assessed using the FACT-Hep (Functional Assessment of Cancer Therapy-Hepatobiliary), a hepatobiliary (liver, bile duct, pancreas) cancer-specific questionnaire comprised of 45 items to specifically assess quality of life. [47] The Brief Fatigue Inventory (BFI) is used to rapidly assess the severity and impact of cancer-related fatigue including six items that correlate with QOL measures. [48] Depressive symptoms are assessed using the 20-item Center for Epidemiologic Studies Depression (CES-D) scale, which was designed to measure the individual’s current level of depressive state. [49,50] Sleep quality is assessed using the Pittsburg Sleep Quality Index (PSQI) which contains 19 questions evaluating seven domains of sleep: subjective sleep quality, sleep latency, sleep duration, habitual sleep efficiency, sleep disturbances, use of sleep medication, and daytime dysfunction. [51,52] The Brief Pain Inventory – short form (BPI) is used to assess the impact of pain on QOL. The BPI includes a sensory and reactive dimension and has been previously validated in breast cancer survivors. [53,54] Barriers to recruitment and exercise adherence are assessed using the 17-item Barriers to Recruitment Participation Questionnaire (BRPQ), [55] and the 43-item Exercise Benefits/Barriers Scale (EBBS). [56] Participant burden is assessed using the 21-item, Perceived Research Burden Assessment (PRBA). [57] Physical function is assessed using the Patient Reported Outcome Measure Information System (PROMIS) Physical Function-10 scale. [58]

2.6.4.Covariates and other measures

*2.6.4.1.Sociodemographic and clinical information*

Sociodemographic are collected using a set of questions self-reported by patients and extracted from medical records. Medical and surgical history is obtained from the treating physician providing clearance to participate in the trial.

*2.6.4.2.Dietary assessment*

Automated self-administered 24-hour dietary assessment tool are used to assess recent dietary patterns for two weekdays and one weekend day, recording all food and drink consumed during the previous 24-hour period. [59] Patients’ consumption of macro- and micronutrients is analyzed.

*2.6.4.3.Physical activity assessment*

Patients are provided with an accelerometer (ActiGraph, Pensacola, FL) to wear for seven days at every testing visit and are instructed to wear the water-resistant accelerometer at all times except for when swimming, showering, and sleeping. The device is provided to the patients, along with instructions of use and postage to return to the study staff after the seven-day period.

**2.6.5. Measures taken during intervention**

*2.6.5.1.Physical activity log and protein supplementation log*

During the exercise sessions, those in exercise groups are asked about any physical activity completed since the last supervised exercise session. Their response is recorded on the exercise session data collection sheet. The AC group completes daily physical activity logs throughout the intervention period. Additionally, those in the resistance exercise and protein supplementation group are asked about the consumption of the protein supplement since last supervised session with whether they did or did not take it. This is also noted on the exercise data collection sheet.

*2.6.5.2.Safety considerations: Questionnaires completed during the exercise sessions*

Exercise tolerance is recorded using the Exercise-Induced Feeling Inventory (EIF). This inventory assesses the individual’s feeling state change(s) post-exercise and is also used for the assessment of self-motivation to adhere to the resistance exercise regimen. [60] Patients are asked to complete the inventory before and after each exercise session. Therapy-related symptom checklist (TRSC) is used to assess and identify patient-reported symptom occurrence and severity each visit for the exercise groups. TRSC contains a 25-item checklist, with response choices ranging from “0” (none) to “4”(very severe). Any symptoms identified as “Severe” or “Very Severe” are discussed with the patient, with potential actions including: no exercise, reduced intensity, removal of an exercise, or an alternative exercise incorporated.

### 2.7. Adverse events

Adverse events (AEs) will be reported and graded according to the revised NCI Common Terminology Criteria for Adverse Events (CTCAE) version 5.0. Investigators must promptly notify the Principal Investigator (PI) of any unanticipated problems or life-threatening complications. Any adverse events occurring after the initial dose of study treatment, during treatment, or within 30 days of the last dose, must be reported to the PI using the local institutional SAE form. All expected and unexpected AEs will be communicated to the PI, who will then report them to the institutional review board. Serious adverse events must be reported within 24 hours of occurrence or discovery. All adverse events, both serious and non-serious, along with any deaths occurring during the study initiation, intervention, and within 30 days of the last study intervention, will be monitored until resolution, stabilization, or until the investigator determines the event to be irreversible, or the participant is lost to follow-up. Adverse events will be documented in the participant’s medical records and the appropriate case report forms. Given the remote nature of the exercise sessions, additional safety measures include providing specific exercise and assessment recommendations (e.g., standing near a wall for balance activities), adapting exercise protocols as needed (e.g., limiting weight or modifying resistance exercises), verifying participants’ home addresses to enable emergency response, and ensuring up-to-date emergency contact information is available for all participants.

### 2.8. Data management plan and timeline of study

Participant recruitment started in September 2022. Currently 18 patients consented, and 19 patients have completed the intervention. Data is monitored internally within DFCI for timeliness of submission, completeness, and adherence to protocol requirements. Monitoring begins at the time of patient registration and continues throughout protocol performance and completion. The study team collects, manages, and performs quality checks on the data. Potential audits or inspections may be conducted by the principal investigator or their designated representatives. All data is stored on a secure network drive using REDCap, a HIPAA-compliant web-based application hosted by Partners HealthCare Research Computing, Enterprise Research Infrastructure & Services, on password-protected computers. Any hardcopy data is stored in locked filing cabinets in card-access facilities.

### 2.9. Sample size calculation

We plan to recruit 45 patients (n = 15 per group). This sample size was determined based on recommendations of samples sizes between 24 and 50 for feasibility studies for assessing the feasibility of the intervention and estimating effect sizes for designing future studies. [61–63] The primary endpoint of this study is feasibility of the resistance exercise combined with protein supplementation intervention. When the sample size n = 15 in the resistance exercise combined with protein supplementation group and the true feasibility rate of 85%, we can observe a feasibility rate ≥70% with the probability of 82%.

### 2.10. Statistical analysis

Based on the intention-to-treat principle, the primary analysis population is all randomized subjects. The secondary analysis population is the per-protocol set (analysis that only includes patients’ data for those who were compliant to the study protocol). The primary endpoint of this pilot study is feasibility of resistance exercise combined with protein supplementation (RE + PS). Using the data from the patients assigned to the RE + PS, we will calculate the point estimate and corresponding exact 95% confidence interval (CI) for the feasibility. For the other outcomes (i.e., skeletal muscle outcomes and biomarkers), we will use standard statistical techniques. We will use descriptive statistics to summarize the data. For within-group comparison (i.e., pre- vs. post-intervention comparison), we will use paired t-test or Wilcoxon signed-rank test for continuous outcomes and McNemar’s test for dichotomous outcomes. For pairwise comparisons between two groups, we will use two-sample t-test, Wilcoxon rank sum tests, Fisher’s exact tests, and so on. ANOVA and logistic regression analyses will be used to explore the association of the baseline characteristics with outcome. For longitudinal data, we will use generalized linear mixed effects models.

The primary analysis includes all completed outcome assessments (regardless of whether patients stayed on the intervention), inviting those who drop from the intervention to return for outcome assessments. The only missing data here would be those who drop the intervention and do not return for outcome assessments; this includes patients withdrawn due to noncompliance. Preliminary analyses will compare patients who do and do not contribute to this analysis on baseline characteristics. A sensitivity analysis will include multiple imputation of all missing outcome data, so that this analysis would be a complete case analysis, with data for all outcome time points.

## Discussion

The primary aim of the REBUILD trial is to evaluate the feasibility of conducting an exercise and protein supplementation intervention (treatment dependent, 16-week maximum) consisting of a home-based virtually supervised resistance exercise and daily protein supplementation in non-metastatic pancreatic cancer patients receiving neoadjuvant chemotherapy. Secondary objectives are to examine the effects of resistance exercise with protein supplementation on skeletal muscle mass, plasma biomarkers associated with tissue wasting (IL-6, MCP-1, TNF-RII, and branched chain amino acids), physical function, and psychosocial measures as compared to RE alone and usual care.

Findings from the REBUILD trial will be highly relevant for the scientific literature, given that this trial targets a vulnerable population of pancreatic cancer patients undergoing neoadjuvant chemotherapy who have been largely underrepresented in previous exercise and nutrition interventions compared with other cancer groups. [64] Furthermore, this trial will contribute to the breadth of existing work exploring the synergistic effects of a multimodal intervention in skeletal muscle loss, sarcopenia, and cachexia-related markers. Within this context, body composition data have highlighted the clinical importance of skeletal muscle on cancer clinical outcomes such as adverse events and toxicities, in recent years. [65] Lower muscle mass before treatment negatively impacts prognosis given its association with chemotherapy toxicity [66,67] and postoperative complications among cancer patients. [68] Particularly, in patients with pancreatic ductal adenocarcinoma, skeletal muscle mass loss soon after diagnosis is a strong predictor of post-treatment complications and survival, [69] likely due to the role of muscle mass in blunting chemotoxicity. [70] Additionally, pancreatic cancer patients with cachexia, showed higher rates of post-operative morbidity (i.e., cardiac and metabolic diseases) [71] and lower post-surgical survival (i.e., 451 vs. 654 days) compared with pancreatic cancer patients without cachexia. [72,73] In support of this finding, cachexia related blood biomarkers such as interleukine-6 (IL-6), monocyte chemoattractant protein-1 (MCP-1), soluble tumor necrosis factor receptor II (TNF-RII) and branched-chain amino acids (BCAAs) are associated with greater muscle loss. [69,74] These biomarkers may provide insights into the inflammatory and metabolic changes associated with cachexia and help identify those pancreatic cancer patients at higher risk of muscle wasting. [69,74]

In this regard, exercise and nutritional interventions emerge as significant non-pharmacological strategies aimed at enhancing skeletal muscle-related outcomes in cancer patients. [10,11,75] Previous exercise oncology trials in this population have shown that exercise was feasible during treatment and had potential to increase quality of life. [76] A systematic review by O’Connor et al. of 12 studies with a total of 300 patients on the role of exercise during pancreatic cancer treatment found exercise to be associated with improved muscle strength, physical function, body composition, fatigue, and quality of life. [77] Bundred et al. evaluated six studies with a total of 193 patients and concluded that prehabilitation programs may improve postoperative outcomes following pancreatic surgery. [78] Furthermore, Luo et al. conducted a systematic review of seven studies and found exercise programs to improve cancer-related fatigue, psychological distress, and physical function. [79] These findings were not without considerable limitations. Most of these trials included single arm non-randomized and non-controlled trials, small sample sizes and very heterogeneous exercise interventions. One previous randomized controlled trial examined the impact of exercise in pancreatic cancer patients receiving neoadjuvant chemotherapy. [80] In this trial, 151 pancreatic cancer patients were randomized to a home-based aerobic and resistance exercise intervention vs usual care during neoadjuvant chemotherapy treatment and found a significant improvement in submaximal exercise capacity in both arms, as well as associations between physical activity and improved fitness, physical function and clinical outcomes. [80]

To our knowledge, the REBUILD trial is the first randomized controlled trial that combines resistance exercise with daily protein supplementation during neoadjuvant chemotherapy in pancreatic cancer patients. While nutritional protein supplementation is not a standard of care for pancreatic cancer patients, it is a strategy recommended by the American Cancer Society as a means to boost energy intake as it has a low impact on the gastrointestinal tract and is high in calories. [81] Another unique aspect of the REBUILD study is the incorporation of a home-based virtually supervised exercise intervention design. The benefits of exercise interventions conducted via virtual supervision are manifold. Said interventions afford an enhanced accessibility, thereby mitigating the constraints of travel time and cost burden. [82] Moreover, home-based virtually supervised interventions facilitate reaching out to patients residing in remote or rural settings, thereby ensuring a broader reach. Furthermore, these interventions enable the preservation of an individualized exercise prescription, a feature not commonly achievable with telephone- or text-based interventions. [82] This exercise intervention design may also be particularly crucial in pancreatic patients due to the complexity of their treatments and side-effects and may reduce barriers to participate in exercise programs for this vulnerable population. [83,84] Therefore, the REBUILD trial is the first randomized controlled trial examining the feasibility and primary efficacy of administering combined home-based virtually supervised resistance exercise and protein supplementation in pancreatic cancer patients receiving neoadjuvant chemotherapy.

A major strength of the REBUILD study is its intervention consisting of moderate-to-vigorous resistance exercise combined with protein supplementation in a vulnerable and understudied population of pancreatic cancer patients undergoing neoadjuvant chemotherapy. Next, patients receive one-on-one virtually supervised exercise training. Home-based exercise sessions virtually administered may facilitate patient adherence to resistance exercise and protein supplementation while ensuring proper exercise techniques. [82] Furthermore, we are using a novel, cost-effective and practical tool to measure skeletal muscle using a fully-automated analysis and standard of care CT scans. [35,36] The REBUILD trial has some limitations that must be acknowledged. One potential limitation is its restrictive eligibility criteria to pancreatic cancer patients initiating chemotherapy may limit generalizability to patients in various phases of pancreatic cancer treatment. Another potential limitation is travel burden associated with testing visits. Lengthy testing visits and numerous questionnaires may result in increased study attrition. To prevent this, testing visits are coordinated with scheduled appointments, and using standard of care measures when possible. Additionally, the REBUILD study population is only representative of the New England region of the US and therefore findings cannot be generalized beyond that regional boundary. The potential adverse events associated with this study include muscle fatigue or soreness after exercise, and rare risks of injury such as muscle strain, ligament damage, or dizziness. Risks of protein supplementation include allergic reactions, mitigated by cross-checking known food allergens with the supplement ingredients. Blood draws carry a minor risk of pain, swelling, or bruising. These risks have been minimized through proper safety measures, including exercise supervision, allergen screening, and standard blood draw protocols.

In conclusion, results from the REBUILD trial will address important research gaps associated with pancreatic cancer-related cachexia and sarcopenia, two conditions closely connected with poor prognosis and mortality. We propose that resistance exercise and protein supplementation in non-metastatic pancreatic cancer patients initiating neoadjuvant chemotherapy will not only be feasible, but also maintain skeletal muscle mass and improve physical function and quality of life. Findings from the REBUILD trial could lead to further investigation into efficacy trials of virtually supervised resistance exercise and protein supplementation in a larger sample size of pancreatic cancer patients, with the ultimate goal of developing future exercise and protein supplementation guidelines during treatment for this population.
